# Case of Emphysema Uteri

**Published:** 1831-05

**Authors:** Charles Mitchell

**Affiliations:** Surgeon.


### EMPHYSEMA UTERI.

### *Case of Emphysema Uteri.* By Charles Mitchell, Esq. Surgeon.

A married woman consulted me, during the month of March, 1830. She had become reduced in circumstances, depressed in spirits, and remarkably despondent; her appetite failed; she became exceedingly nervous and indolent, with the catamenia reduced in quantity, and accompanied with increased pain, to which she had been always, more or less, subject during the menstrual periods. An occasional aperient was administered, with a fourth part of the following mixture four times a day: ℞. Subcarb. Ammon. ʒss.; Inf. Calomb., Mist. Camphor. *aa* ℥iv. M.

She took the medicine upwards of three weeks, without the least amendment, and now, added to these evils, succeeded a most loathsome malady, by which she became a burden to herself, and, to use her own expression, "a nuisance to society:" I mean the collection, and consequent explosion, of air from the cavity of the uterus. A graduated series of compresses were placed over the orifice of the vagina, as might be supposed, indeed, with no beneficial result; for they rather aggravated, than allayed the evil. A piece of sponge was afterwards introduced, with no better success.

Particular attention was now paid to the general health, by daily but moderate exercise, by the allowance of four ounces of wine and six ounces of animal food; while, at the same time, she took six ounces of the decoction of bark, with one drachm of diluted sulphuric acid, in the course of the day, for a whole month; and, subsequently, the muriated tincture of iron. A pint of cold water was also dashed upon the lower part of the abdomen and upper part of the thighs, morning and evening. The general health improved, but the disorder of the uterine system remained stationary, which wounded the delicacy of her feelings, and produced a great degree of restraint upon her public appearance.

By my advice, her removal to the country was readily acceded to: she returned in the course of a month, with considerable increased renovation of her general health, but unrelieved from her harassing and loathsome malady, by which she was apparently "doomed to pass a miserable existence," neither being caressed or "cheered by the least ray of hope," but afflicted by a poignant feeling of everlasting seclusion from her circle of society. I, however, anticipated some advantage might result from the introduction of an elastic gum catheter into the cavity of the vagina, where it was secured by tapes; and, in the course of a few days, I had much pleasure in finding she had obtained some benefit from its introduction, the evil, according to her own judgment, being in some degree removed. The result, however, did not altogether realize her expectations, and I was consequently obliged to pass the instrument through the os uteri, where it was introduced, and retained with much difficulty, as it slipped out at every movement, until it was secured more firmly by tapes twisted in every direction. A horizontal position was strictly enjoined; the irritation, comparatively speaking, was trifling, and in the course of a very short time subsided. The explosions were completely subdued, and she could move about without annoyance, unless when the apartment was heated. She removed to Lambeth about October, and I have not seen or heard of her since.

I conceive a ball pessary, with an elastic gum tube proceeding from its extremity, would be very efficacious in similar cases; and I shall not fail to give it a trial, if such a case come again under my observation.

*Lamb's Conduit street; April 4th,* 1831.

